# Assessing the Storage Root Development of Cassava with a New Analysis Tool

**DOI:** 10.34133/2022/9767820

**Published:** 2022-10-26

**Authors:** Jens Wilhelm, Tobias Wojciechowski, Johannes A. Postma, Dirk Jollet, Kathrin Heinz, Vera Böckem, Mark Müller-Linow

**Affiliations:** ^1^ Institute of Plant Sciences, IBG-2, Forschungszentrum Jülich GmbH, 52425 Jülich, Germany; ^2^ Tesla Automation GmbH, 54595 PrümGermany

## Abstract

Storage roots of cassava plants crops are one of the main providers of starch in many South American, African, and Asian countries. Finding varieties with high yields is crucial for growing and breeding. This requires a better understanding of the dynamics of storage root formation, which is usually done by repeated manual evaluation of root types, diameters, and their distribution in excavated roots. We introduce a newly developed method that is capable to analyze the distribution of root diameters automatically, even if root systems display strong variations in root widths and clustering in high numbers. An application study was conducted with cassava roots imaged in a video acquisition box. The root diameter distribution was quantified automatically using an iterative ridge detection approach, which can cope with a wide span of root diameters and clustering. The approach was validated with virtual root models of known geometries and then tested with a time-series of excavated root systems. Based on the retrieved diameter classes, we show plausibly that the dynamics of root type formation can be monitored qualitatively and quantitatively. We conclude that this new method reliably determines important phenotypic traits from storage root crop images. The method is fast and robustly analyses complex root systems and thereby applicable in high-throughput phenotyping and future breeding.

## 1. Introduction

Belowground storage crops (tuber and root) like cassava, sweet potato, taro, yams, or potato are an essential source of starch, and other carbohydrates thereby not only contributing to food security worldwide but also providing diverse and important byproducts [1]. The majority of these crops is grown in Africa, followed by Asia and South and Central America, while in Europe only potato and sugar beet are grown frequently [2, 3]. The genetic and phenotypic characterization of root and stolon morphology and root architecture represents one of the biggest challenges that needs to be solved in order to provide a knowledge base for future breeding, crop management, and yield [4]. The formation of the storage roots is one of the key processes that needs to be addressed more comprehensively. In cassava plants (*Manihot esculenta* CRANTZ), these morphological changes are caused by swelling, where fibrous roots have fast secondary growth (thickening). An overview on different studies focusing on this process is given in [5, 6]. A temporally resolved characterization of root systems, particularly focusing on storage root formation and root classification is foundational to cultivar selection and breeding. In cassava, the number of storage roots varies between 3 and 10 [7]. Besides storage roots, Gregory and Wojciechowski [5] distinguish 3 other root types, which are type I: fibrous roots (AR), type II: transition roots (TR), type III: early storage roots (ESR), and type IV: storage roots (SR). This subdivision is based on genetic expression profiles, but also related to typical maximal diameters for each type. For the evaluation of a specific genotype, the distribution of root types at a given time point is an important indicator of root system functioning and, in the case of storage roots, yield. Measuring root traits and classifying roots is challenging, especially when high-throughput is required under field conditions. In situ systems would be the preferred option, because changes of root system architecture can be monitored continuously. However, such approaches exhibit different limitations, which either arise from restricted spatial observation options like in rhizotron systems [8] and imaging tubes [9] or from a spatial limitations for root growth, which are present in flat growth containers like rhizotrons or paper pouch systems [10], or in restricted diameters of growth tubes, which are used for tomographic methods like NMR [11] and CT [12, 13], the latter ones also being quite costly. With respect to storage root crops, the majority of these approaches is not designed for larger root diameters, and therefore, they are only capable to cover the first developmental stages (e.g. a single cassava root may reach a diameter up to 15 cm). An approach applicable but also restricted to larger roots is based on ground penetrating radar as demonstrated for tree-like roots in [14].

An alternative and common practice is the excavation of root crowns in their natural growing environment, and the subsequent measurement of root traits, also known as ‘shovelomics’ [15–17]. Even though the continuous monitoring of individual root systems is not possible, the developmental progress may be measured based on population data. Taking measurements of root system architecture traits by hand is increasingly replaced by (semi) automated methods. Success of automation varies by trait of interest, e.g. width, length, covered area and density, as well as root architecture traits like rooting angles, root length, and specific root length (see e.g. Win-Rhizo [18, 19], DIRT [20, 21], GIA-Roots [22], and REST [16]). These software developments typically only work for images taken in very specific contexts (species, image resolution, (back) lightning, etc.). Particular to storage roots, Atanbori et al. [23] were able to identify and count storage roots of cassava plants by training a convolutional neural network. Seethepalli et al. [24] imaged the root system in a box with backlight illumination. This increases the contrast between root systems (black) and background (white). They identified root centers with a skeletonization method [25] based on black-white images, which is a common approach for root center line identification. In contrast to other approaches, the authors also tried to assess the diameter of single roots.

This approach is restricted, however, to root systems, where the imaged roots show some displacement by the background. The limitation comes in denser root systems or in root systems, where larger root diameters lead to a stronger clustering and where interspaces between roots vanish in the 2D representations. This is the typical scenario in storage root crops like cassava, where roots are often located side by side.

With this manuscript, we present a new method for the quantification of a number of structural root traits of storage root crops, using cassava root systems as a test case due to its economic importance. In particular, we focus on the tubular diameters of various root types. Our approach is suitable to analyze videos that capture the root from all sides. It consists of an image processing pipeline that includes two essential parts: the first one consists of segmentation procedures for roots, background, and stem, the latter one via a neural network approach; the second part includes the detection of single roots or root sections and the analysis of their diameters. Here, we developed an iterative version of a ridge detection approach, which was introduced by Steger [26, 27]. In this way, we were capable to identify and measure roots even in strongly clustered root crowns. An essential part of this work is the validation of the pipeline with virtual root models from single roots to complex root systems. The models were simulated with the OpenSimRoot (OSR) software [28] using a parametrization that emulates characteristics of cassava root architecture and growth, in particular typical root diameter distributions at different growth stages, which cover ranges between 1 mm and a few cm. To evaluate our approach on real root systems, we designed a box for video recordings and analyzed the video frames with the parametrization that worked best on our artificial images. We show different examples that highlight the capability of our software pipeline to resolve root diameters of different root types at different clustering levels allowing a high-throughput phenotyping for future breeding.

## 2. Materials and Methods

### 2.1. Cassava Root Material

Cassava (*Manihot esculenta* CRANTZ) was used to investigate storage root development due to its economic importance providing carbohydrates to approximately 800 million people mainly in the tropical and subtropical regions. We focused on the formation of storage roots between week three to twelve developing this software. All plants (greenhouse and field) derived from stem cutting of a length of 20-30 cm. The figures with imaged roots in the last sections and supplements give a good impression on the contrasting root architectures and different developmental stages. We phenotyped fibrous root development over time, which were classed based on gene expression and diameter into different root types: fibrous, transition, early storage, and storage (fibrous) roots, which correspond to the following maximum diameter: type 1: <2 mm; type 2: 2-6 mm; type 3: 6-20 mm; type 4: >20 mm, respectively. If we refer to the diameter classes in the following, it has to be delineated from the root types. We define a diameter class as a particular range of diameters independent of the root type. A more mature root, for example, is likely covering more than one diameter class. From the geometrical point of view of our analysis a root type is therefore defined by its highest diameter class. We split up the diameter classes according the ranges that were used for the root type definitions, for example, finding a diameter class 4 points to the existence of a storage root (type IV). The distribution of diameter classes is therefore defined as the different ratios of each class with respect to their constituting lengths.

For the single picture, the cassava variety Kasetsart University 50 (KU50) was grown in the greenhouse facilities of the Institute of Bio and Geosciences (IBG-2) at the Forschungszentrum Jülich GmbH, Germany (50° 54′36 ${}^{\prime \prime }$N, 6° 24′49 ${}^{\prime \prime }$ E). A loam field soil (10% clay, 38.6% silt, and 51.4% sand) was used for cultivation. The plant cuttings grew in the following conditions: 29°C/24°C (day/night), 70% relative humidity, light 14/10 h (day/night), and a light intensity of ≥600 *μ*E/s x m^2^ for 8 months.

Field grown plants grew in Rayong Field Crop Research Center (12°44′01${}^{\prime \prime }$N, 101°08′02${}^{\prime \prime }$E) up to twelve weeks. The plants were grown in loamy sand soil. Although plants were grown in different seasons, they were irrigated to maintain similar well-watered soil conditions. We harvested six plants of each genotype each week between the third to the twelfth week.

### 2.2. Imaging Setup and Video Acquisition

The image of an immobile root system in front of a black background was acquired with a Canon 5d Mark II using a reduced resolution of 3000×2000 pixels. The image was further cropped to 1615×1393 pixels displaying the complete root system. The video imaging setup consists of a closed blue-colored box that contains a turnable bracket for root fixation in the center of the ceiling, artificial lighting, and a DSLR camera (Canon 80D), which is mounted lateral to the root system in a distance of 113.5 cm. The illumination is provided by 4 LED panels that are fixed beside the camera. The bracket is connected to a step motor, which is operated by a control software that synchronizes rotation speed and camera triggering. The camera was operated in video mode with full HD resolution. The focus was fixed to the distance to the rotation axis. Video frames were captured roughly every 2°, and videos were finally stored in the *mov* format. In addition, the box allows detection and decoding of QR-code labels, and the content is automatically attributed to the processed data results. For comparison purposes we also acquired images outside the video box to get root pictures without the blurring effects from the rotation motion.

### 2.3. Video Processing

**Figure 1:**
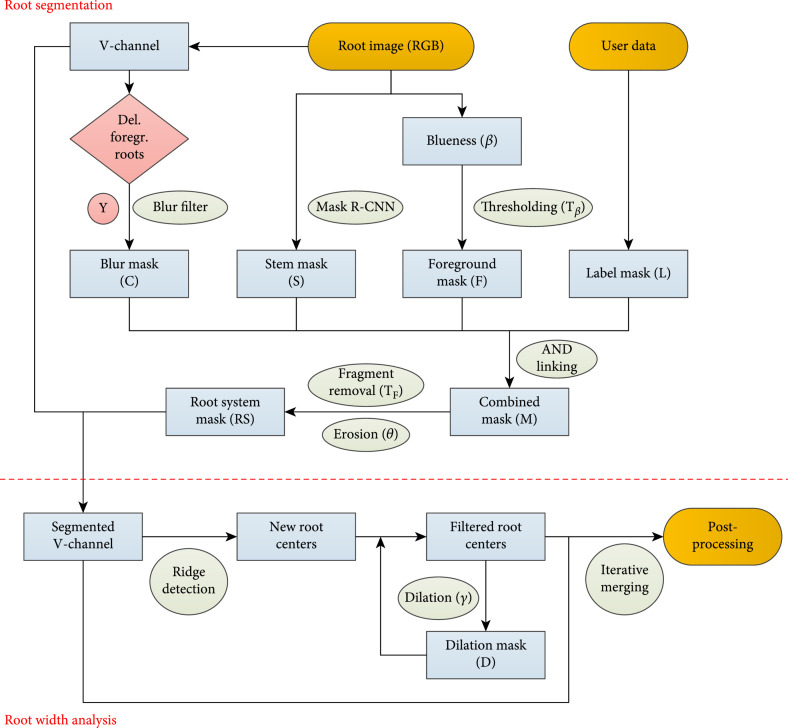
Flowchart of the image processing pipeline for a single image or video frame. The workflow is split up into two algorithmic modules (separated by the red dotted line) for root system identification (segmentation) and for the computation of the root diameter distribution (width analysis).

The software was written in C++ (Service/Backend) and C# (GUI/Frontend). It runs under Windows and requires a graphics card with CUDA 11.2 support and a Halcon Licence [29]. It allows the import of both image sequences (in the tagged image file TIF format) and videos (in the MOV format). All image sequences presented in this study were processed on an Intel i7-4770, 3.40GHz CPU and an Nvidia GTX 1080 graphics card. The processing for a sequence of 180 images took ~10 min. The image processing pipeline for the analysis of a single image or video frame is subdivided into two larger modules; an overview is given in Figure 1.

The first module *Root Segmentation* contains semantic segmentation procedures for the stem (subsection *Stem detection*), the root system architecture (subsection *RSA segmentation*) and additional targets like QR-code labels. The image position of a QR-code is provided by the user. The QR-code content is automatically decoded by the software and used to reference the results. The second module *Root Width Analysis* applies a ridge detection approach [30, 31] on the outcome of the segmentation thereby identifying root centers and analyzing the width distribution of the root system architecture (subsection RSA analysis). The *Root Width Analysis* module is not capable to extract ridge-related features of roots correctly from the original RBG image data without masking the background. Firstly, it is required that the background does not contain any biasing patterns that could lead to wrong detections, e.g. the corners of the imaging box. A much more important role, however, plays the space between neighboring roots. Because many roots follow a similar path, the space between may also have elongated shapes, which are then wrongly attributed to root-like structures. Instead of removing these false positives in a post-processing step, it is important to remove the background prior to the *Root Width Analysis* due to an additional unwanted effect, which prevents particular roots from being detected.

#### 2.3.1. Stem Detection

Roots systems are fixed with their stem residues to a mounting bracket of the box. Because stems are cut at different positions, root systems were hanging down at different box levels. Therefore we decided to adjust the camera such that the root system is captured completely including the stem. In order not to be misclassified as thick roots, we need to remove the stem from all images, which is done in the first module (Figure 1, top). For this semantic segmentation task we used an artificial neural network and trained it with 210 annotated images (split up into 168 training and 42 validation images). For annotation, we marked all stem borders with closed polygonal chains. The rather small number of training images proved to be sufficient for this task. As network model we used a Windows-compatible version of the Mask R-CNN [32] from the Detectron2 suite [33], which is Facebook’s framework of state-of-the-art algorithms for different object classification and segmentation tasks. To achieve a sufficient throughput speed we selected the Mask R-CNN R50-FCN 3× architecture, which contains a 50-layer ResNet (R50) Feature Pyramid Network (FPN) backbone [34]. This model makes a good tradeoff between prediction accuracy and speed [34]. The model used pretrained weights from ImageNet with the standard configuration of hyperparameters [33] except for the following settings: batch size was limited to 1, training time: 100000 iterations; learning rate: 0.00025. For inference, the trained model was applied with the standard set of parameters as well. Segmented stems in the prediction are represented as ones. For further processing, the results were inverted such that stems pixels got zero values (stem mask S).

#### 2.3.2. Root System Architecture Segmentation

Subsequent analysis depends on the correct segmentation of the root system (RS) without stem, further objects and blurred roots. The stem mask S is computed as described in the previous subsection. Additional targets like QR codes have fixed positions (provided by the user) and get zero values in the binary label mask L (Figure 1, top). Blurred roots may emerge in video sequences due to the rotary motion of the root system and strongly affect the width estimation as they appear broader than they are. These roots are identified with a Canny edge detection filter [35] and are represented as zeros in the binary blur mask C (Figure 1, top). These masks are finally combined together with a binary representation of the foreground F resulting in a binary mask M, which may be further refined by morphological erosion (with erosion width θ) to the root system mask (RS). Merging the masks is carried out via logical AND operations as follows:

(1)M=F∧S∧L∧C.

The segmentation of the foreground F takes advantage of the contrasting blue background of the imaging box. Instead of detecting the root system itself, we applied a thresholding operation on a normalized blueness filter to detect the background. The blueness filter is adapted from the ExGR greenness filter, described in [36], including the proposed channel weights. The blueness β is calculated from the image RGB channel values:

(2)β=3B−2.4G−R.

Pixels with normalized blueness values $\beta^{\prime }=255*\beta /\max\beta$ below a user-defined threshold $T_{\beta }$ are considered as foreground F. The removal of blurred roots is based on the fact that motion blurring (a video imaging effect) prevents roots from being displayed with discernable edges. Therefore, a canny edge filter is applied to detect roots with distinct edges. These edges are enlarged by morphological dilation operation, and the result is the blur mask C with zero-valued pixel that are considered blurred roots or background. Before RS is used to mask the gray value representation of the RGB image small fragments below a pixel threshold $\;T_{F}$ may be filtered.

#### 2.3.3. Root System Architecture Analysis

Single root detection together with diameter measurements at any position along the root center line (RCL) is based on a method introduced by Steger [27], which we extended to deal with various combinations of root widths within one image. A detailed overview on the method is given in [27]. Here, we only focus on the scientific background, which is necessary to understand the parametrization of this approach. The width analysis of curvilinear structures is based on the detection of the central root axis (ridge detection) together with the corresponding edges. The image is regarded as a function  f, which in turn can be analyzed via differential geometry by means of a sliding convolution filter  c, resulting in a response r.

(3)r=c∗f.

Nonartificial images are usually suspect to noise, which complicates the analysis. In our case the root systems display heterogeneous surface patterns, different colors, and dirt particles. Therefore, the method uses discrete Gaussian masks of first and second degree to smooth the image data on the one hand and to detect ridge centers (low responses in the first derivative) with high curvature (strong responses in the second derivative) on the other [26]. In the following, we restrict the description to the 1D-case, which is sufficient to understand the principle of the parametrization. The shape and consequently smoothing strength of the Gaussian kernel is determined by the smoothing parameter σ as illustrated in the 1D-Gaussian density function:

(4)gσx=12πσe−x2/2σ2.

Consequently, parameter σ also determines the maximum root diameter, i.e., higher values of σ are required to cover larger diameters. As long as imaged root diameters stay within a particular range, the method works well, but as soon as the diameters deviate distinctly the method becomes less accurate, which affects the correct estimation especially of smaller root diameters. Because parameter σ needs to be tuned to the largest diameter in the image, this may result in an overblurring of smaller objects, i.e., small roots become almost erased. Parameter σ needs to be adjusted according to the maximum root diameter half width  w, such that $\;\sigma \geq w/\sqrt{3}$ [27]. The third parameter  h reflects the image contrast between root pixels and background pixels. Parameters  w, σ, and h are required to estimate a threshold u for the signal response $\;r^{\prime \prime }$ at the center position of the root (x=0 in the 1D-representation), when convolving the image with the second derivative of the Gaussian kernel analogous to Equation (3):

(5)r′′x=0,σ,w,h=g′′x∗fx=hg′w−g′−w=2wh2πσ3e−w2/2σ2.

The threshold u is used to identify starting points on a line that represent a ridge. The method employs a second threshold  l, which lies below threshold u and which is required to fill in line points between starting points. Regarding the ratio between lower and upper threshold no recommendation is given in [27]. The method is also part of the machine vision software Halcon [29], which suggests a range between 0.25 and 0.5. The position of the edges, which is required to compute the width distribution, is given by the maxima, when convolving the image with the first derivate of the Gaussian kernel. This corresponds to the zero-crossings of $\;r^{\prime \prime }$ (see Equation (5)). The 2D-image case requires to include the orientation of the RCL into the edge point localization. Therefore, the maxima of the absolute gradient values perpendicular to the line need to be computed. Every center line point requires at least one edge point on each side in order to compute pixel-wise width values. The edge computation and also the interpolation method for missing edge points is explained in detail in [27].

Another important feature of this method is the invariance against different contrast levels along both sides of root edges. It is impossible to guarantee a homogeneous illumination at any location in the imaging setup. However, inevitable differences in contrast levels have only a marginal impact on the positional accuracy of the RCL. As indicated before, the method is not capable to locate every root and to analyze the respective root widths correctly, if a wide range of root diameters is present. Therefore, we extended this approach by iterating through a number of parameter constellations, where the number of iterations depends on the maximum root width. It is clear, however that just merging the results from different iterations could lead to several proposals for the RCL and consequently root widths. Therefore, one needs to take care that the line proposals from subsequent iterations extend the old ones without overwriting them. This is ensured by a dilation mask D in our implementation that is computed from the previously identified iteration (except for the first) by morphological dilation of the skeleton of root centers. The dilation masks are adding up in every iteration. The kernel width γ of the dilation depends on the parametrization of the ridge detection for the current iteration. It should not be higher than the respective root diameter $\;\left(2w\right)$; we applied $\;\gamma =0.9*\left(2w\right)$. The deletion mask D preserves the respective pixels from being used as ridge line pixels in the next iteration. New root centers can only form in a certain distance to the lines that are already in place.

Another issue is the selection of the iteration direction, as it is possible to start with the detection of the smallest root diameters (*forward*) or with the largest (*reverse*). The *reverse* option requires an additional parameter  τ, which limits the minimum diameter in each iteration. Otherwise, detected root centers from the first iterations would prevent further root centers to be found in the latter iterations. We tested both versions and both seem to be valid and the better option depends on both image content and on the main target that needs to be analyzed in the image. If the focus is only on roots with larger diameters, the *reverse* option is most likely the better choice. For the analysis of the OSR models and the video sequences of real cassava roots, we used the *forward* option as the test case of a single root image (taken outside the video box). We used the *reverse* option since this sample is featured by many ESR and SR root type. The outcome of this iterative process is an image of root center pixels, where each pixel is associated with a respective root width value.

However, in order to produce reliable statistics from this output further postprocessing steps are required due to the following reasons. Firstly, the ridge detection algorithm identifies RCLs with different pixel neighborhood properties. Especially, in thin roots incomplete root centers with regular pixel gaps may occur. In larger roots, the center line sometimes appears broader. In these cases, some pixels have up to four neighboring pixels (assuming an 8-pixel neighborhood scheme) instead of two, which would corresponds to the thinnest representation of a line (without junctions). Secondly, in some cases, RCL pixels may be detected between roots (i.e., in the background), if roots are parallel arranged and misjudged as one thicker root. Another factor is the orientation of line segments. A pixel in a vertical or horizontal segment represents a smaller metric length than a pixel in a diagonal segment, which metric length would be underestimated by up to ~29%. Therefore, we applied the following postprocessing steps to account for these biasing factors. In the first two steps, morphological operations are applied on the output; lines are dilated by one pixel to close gaps of one pixel size followed by a skeleton extraction thereby thinning all RCLs to their minimum width. The resulting map is filtered with the root system mask RS to remove RCLs in the background. In the last step, the orientation of RCL segments is estimated with a HOG (histogram of oriented gradients) features descriptor [37]. The image is partitioned into small sections, and each is featured by a histogram of gradient strengths for a predefined number of orientations. From the dominant orientation $\omega_{R}$ [rad] a length correction factor τ for the RCL pixels of the respective segment is computed according to$\;\tau =\left|1/\cos\left(\omega_{R}\right)\right|$.

#### 2.3.4. Output

The main output of our approach is a list of pixel coordinates with corresponding width values in pixel-based and Euclidian metrics (mm) for each analyzed image. The conversion is computed for the distance between camera and axis of box’s rotary system. In case of our simulated validation data, each image represents a different root system (or single root). This would also be true for the real case, where root growth and consequently imaging is limited to 2D-space like in paper pouch or Rhizotron systems. For our use case of cassava plants, we captured the root system from all sides in a video box to give each root the opportunity to be in full view, because each image represents only a fraction of the root system. For the statistics in the study with real roots (section 2.5), we simply averaged the diameter distributions over all perspectives. Beyond that, we assume that some of the variation coming from the different perspectives and occlusions is hereby removed. Another option would be to take the maximum over all perspectives for each diameter class. However, we did not investigate the multiview aspect further, because for our application scenario, it was sufficient to know when a particular root type emerges and to what extent. Furthermore, the complete image sequence may be used for the computation of other RSA parameters like RS width or length.

### 2.4. Validation Using Simulated Root Systems with Known Diameter Distributions

OpenSimRoot is a software for simulating root systems in 3D [28]. We used OpenSimRoot to simulate cassava root systems which visually looked similar to the excavated root systems when imaged from the side (like in a fronto-parallel camera-root setup). Root classes, their diameters, and their secondary growth were simulated based on previous measurements. OpenSimRoot simulates the growth of entire root systems in 3D. However, we restricted growth to a 60×150×0.6 cm volume to make sure that roots grew perpendicular to the viewing direction. From each resulting model, 2D images were rendered using Paraview (http://www.paraview.org). Paraview can filter out individual roots, and each root was rendered individually, using a python script. The rendering was from a side view which displayed the largest expansion of the root. Given that the roots grew perpendicular to the viewing direction, there was little perspective shortening. This constrained was necessary to enable a direct comparison between the root width distribution provided by the simulation parameters of the model and the one analyzed with our approach. We started with the simplest validation scenario using single roots of varying root types and continued with root systems that we assembled from individually rendered roots.

For both validation experiments, we simulated the growth of 5 different root systems (each consisting of 80 single roots) over a time span of 13 weeks. A rendered version of each root together with the distribution of root diameter classes, according to the OpenSimRoot scheme, was stored at the end of each week. The rendering included a front light similar to the imaging box used for the real roots. This directed light gave the roots a shading and thereby round impression sim. The render script also wrote the rendered root length (mm) in the following diameter classes: 0-1, 1-2, …, 9-10, 10-15, 15-20, 20-25, 25-30, and 30+ (mm). For validation purposes, classes in this distribution were merged to fit the four diameter classes I to IV that are used for cassava root classification [5]. An important constraint of the imaging system required additional modifications; with respect to the smallest root type (<2 mm), the image resolution of the setup was not sufficient to deliver reliable results. To test the feasibility of our analysis pipeline, we therefore rendered single roots for the first validation study such that the minimum diameter of 3 pixels corresponded to diameter class I. For the remaining tests, we used rendered root models with a resolution comparable to the real imaging box setup, taking into account that class I roots were displayed with 2 pixel or less and therefore not detected or classified correctly.

**Figure 2:**
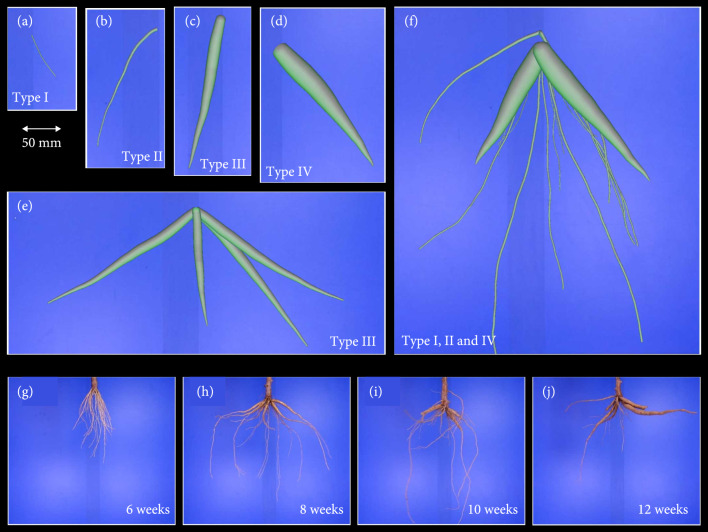
OpenSimRoot models and real root systems. (a–d) Single roots of type I (fibrous), type II (transition roots), type III (early storage roots), and type IV (storage roots). (e) A simple root system consisting of early storage roots only; (f) a more complex root system consisting of three root types; (g–j) 4 different root systems excavated in 2-week intervals and imaged with the video acquisition setup.

For the first validation experiment, the generation of single virtual root models was carried out as follows; from each of the 5 simulated root systems, 10 roots with a predefined maximum diameter were randomly chosen to generate a validation data set of 50 single roots for each cassava root type I-IV. The maximum diameter was limited to 45 mm. Figures 2(a)–2(d) show one example for each root type. The second part of the validation experiment with more complex root systems was split up into two tests. We analyzed root systems that only contained one root type (Figure 2(e) shows a type III root system). In more developed root systems, this may happen, either due to the excavation itself and the associated loss of small roots or due to senescence of fibrous roots that takes place, if they do not develop further [5, 38]. Furthermore, we simulated the growth of root systems (each with 80 roots) over the entire time span of 13 weeks and analyzed the different growth stages of root subsets in one week intervals. The initial root system served as a building set for the construction of smaller root architectures in order to get a wide range of geometries. To achieve this, 25 root systems were created at each time point by randomly selecting 17 roots from the entire set of 80 roots and removing the rest from the output. The roots were selected such that the proportion of each root type did not change. Thus, we assembled 25 different root systems each week and analyzed them with our pipeline (a root system of week 13 is shown in Figure 2(f)). We did not consider adding a stem to each system, because the stem detection was evaluated separately. In the final step, we added the background (displaying a blue wall like in Figure 2) from the imaging box to all images. Figures 2(g)–2(j) show typical samples of real cassava root systems that were imaged with the video setup. The three root types I, II, and III are also clearly visible. Root type IV just started to emerge after week 12.

### 2.5. Real Root Growth Dynamics from Regular Sampling

With our final experiment, we want to investigate whether our approach is capable to plausibly track the secondary thickening of roots over time. Therefore we selected one cultivar from the harvested plants (grown at the Rayong Field Crop Research Center) in order to analyze the developmental dynamics for each root type, where we would expect a smooth increase for the storage root types III and IV. We chose 8 harvesting dates (between week 5 and 12) and randomly selected 3 root systems each date summing up to 24 video captures, each with 180 frames. To illustrate the growth dynamics, we first computed the diameter distributions of each frame and plant separately and finally combined all results.

## 3. Results

We applied different parameter constellation for the analysis of virtual and real root image and video data due to the different nature of virtual and real images. Table S1 gives a summary of the three principal parametrizations. In the following sections on virtual root models, we analyzed images with the parametrization given in Table S1 (left).

### 3.1. Validation Study with Single Virtual Roots

**Figure 3:**
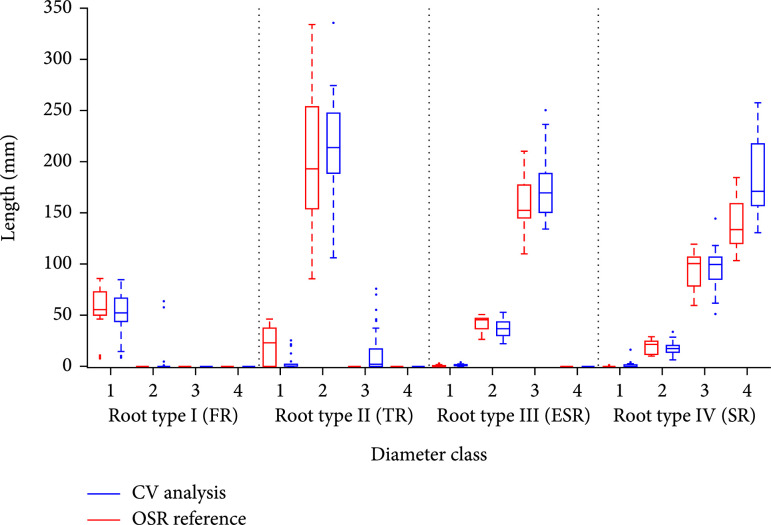
Validation with single simulated root models. For each root type (I-IV), the constituting lengths of all diameter classes (1-4) of the OpenSimRoot Simulation (red) and computer vision prediction (blue) were compared against each other.

For the first validation experiment, the generation of single virtual root models was carried out as follows: from each of the 5 root systems, 10 roots with a predefined maximum diameter were randomly chosen to generate a validation data set of 50 single root images for each cassava root type I-IV. Compared to the other validation studies, these images were computed at a higher resolution to show the capability of the analysis pipeline also to resolve roots <2 mm (type I). We analyzed the statistical dispersion of constituting lengths of each diameter class separately and repeated this for every root type. The results are summarized in the box plot diagram in Figure 3. The root type determines the maximal diameter class. Therefore, the OpenSimRoot (OSR) reference (red) does not display any values for a class exceeding the respective root type. Comparing reference values and analysis predictions, one observes a good congruence of the medians and interquartile ranges, i.e., all root types and their constituting diameter class lengths are quantified almost correctly. The results are supported by a regression statistics (Figure S1(a)) with $\;{\text{R}}^{2}=0.95$ and nRSME=0.27 (the normalization was computed from the standard deviation). One distinct deviation is given for class 4 in the storage root type IV, which results in an overestimation of 13.1% for this diameter class and 5.6% for this root type. An additional look at the imaged output of the detected root center lines (data not shown) revealed a fan-like partitioning of diameter class 4 lines at the root base. Another peculiarity is visible in root type I and II. Here, we observed some predictions of the respective next higher class, although this class should not exist.

### 3.2. Validation Study with One-Root-Type Systems

**Figure 4:**
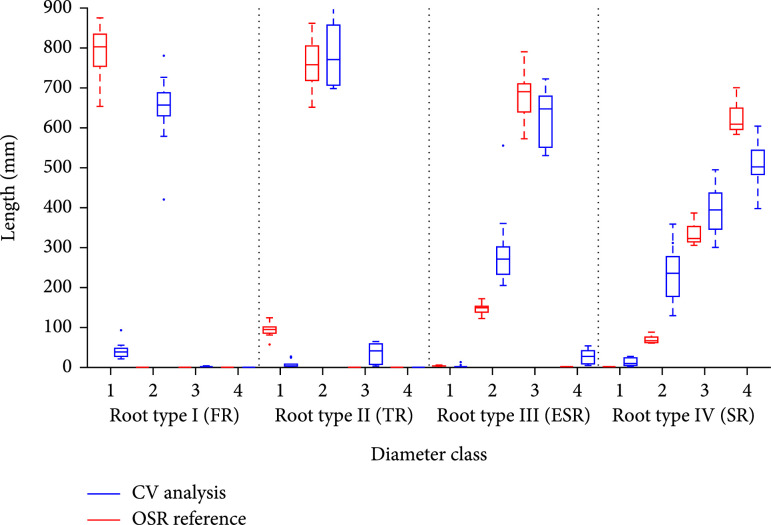
Validation with simulated one-root-type systems. Analogous to Figure 3, the constituting lengths of all diameter classes (1-4) of the OpenSimRoot Simulation (red) and computer vision prediction (blue) were compared against each other.

In the next validation experiment, we testes the analysis pipeline with root system models of reduced complexity (Figure 4). Each root system consists of one root type (Figure 2(e) shows a root system with type III roots). This test is an analogous to the first one, but with more roots that are also subject to self-occlusions. This time, the image resolution was reduced and adapted to our video acquisition scenario with real roots. The statistical analysis was done as described. It is first of all apparent that diameter class 1 is not detected correctly any more (most obvious for root type I). These roots have a diameter ≤2 pixels and are predicted as class 2. Apart from this, the prediction of constituting class lengths shows a similar pattern to the first test, with the difference that reference medians and predicted ones differ a little more, still showing a strong relation. The regression statistics (Figure S1(b) showed an ${\text{R}}^{2}=0.9$ and nRSME=0.31. Here, we considered only the root types II, III, and IV. Interestingly, class 4 roots (in root type IV) are, this time, underestimated. In this artificial setup, the root bases are overlapping near the stem region.

### 3.3. Analysis of a Virtual Time Series of Root Systems

**Figure 5:**
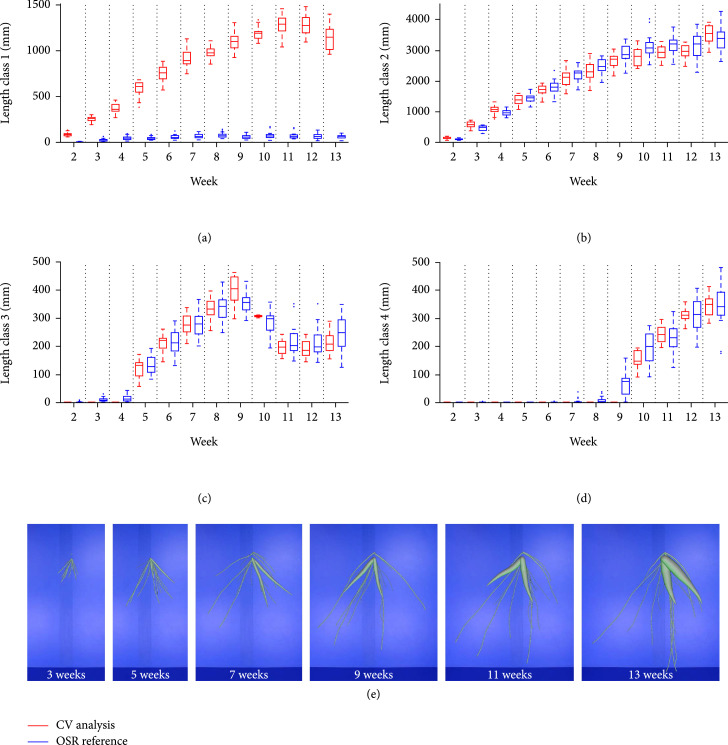
Validation with simulated time-series of root system growth for different diameter classes. (a) Class 1 diameters are strongly underestimated due to the poor image resolution of fibrous roots. Indeed, an increase over time is also indicated in the analysis, but the results do not reflect the absolute values of the simulation. (b, c) Class 2 and 3 diameters display a high concordance between simulation parameters and analysis results at every growth stage. (d) Class 4 diameters appear in week 10, however the analysis detects them in week 9 for the first time. This corresponds to an overestimation of class 3 diameters in the same week (c). In (e), examples of the underlying OSR models at different developmental stages are highlighted.

In the last validation test, we first simulated the growth of a root systems with 80 roots between week 2 (first short fibrous roots) and week 13 (at least 2 storage roots, see Figure 2(f)). Each week, we sampled 25 random subsets each with 17 roots such that the proportion of root types was preserved. Again, we used a reduced image resolution for image analysis to ensure comparability to the real root analysis. The results were analyzed by week and compared to the ground truth root parameters of the OSR models. The growth of the different root diameter classes (Figure 5(a): class 1, (b): class 2, etc.) is indicated by the red (model) and blue (analysis) boxes. As expected, thin root sections with a diameter ≤2 pixel (class 1) are not detected in most cases and the values stay rather constant at a low level (Figure 5(a)). Results for diameter class 2 and 3 (Figures 5(b) and 5(c)) display a high accordance between the OSR ground truth and computer vision results. These results are supported by a regression statistics (Figure S1(c)) with ${\text{R}}^{2}=0.98$ and nRSME=0.15. This finding applies to diameter class 4 as well with one exception; at week 9, a fairly small fraction of roots (most likely of diameter class 3) is wrongly classified as class 4.

### 3.4. Validation of the Stem Detection Approach

**Figure 6:**
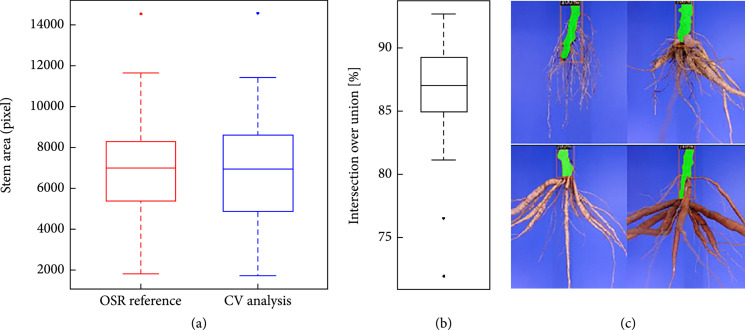
Stem detection with the Mask R-CNN. (a) Comparison of ground truth (reference) and predicted (CV analysis) stem areas displayed an average deviation of 239 pixels (~5% error). (b) The intersection over union (IoU) between ground truth and prediction was ~87%. (c) Four examples of root systems of different developmental stages displaying the high quality of the stem detection (predicted stems are orange-colored).

For the validation of the stem detection results, we used two measures to compare ground truth and prediction, namely, the stem area (pixels) and the intersection over union (IoU), which calculate the percentage overlap of annotated and predicted mask. Figure 6 summarizes the results for 42 images that were not used for training. Figure 6(a) shows a strong match between true and predicted stem areas with an average deviation of 239 pixels, which corresponds to an average error of 5.0%. This is supported by an average IoU value of 87% (Figure 6(b)). Only 2 images displayed a lower overlap (IoU<80%). In Figure 6(c), we show 4 typical examples, which support the good statistics of this approach.

### 3.5. Analysis of the Root Diameter Distribution of Physical Cassava Root Systems

To illustrate the transferability of our approach to estimate root diameter distributions in real roots, we first give an example of an analyzed cassava root image that was acquired outside the video box using the camera’s imaging mode and keeping the root in a fixed position. In the second part, we show examples of analyzed samples of a cassava experiment, where 24 root systems (same genotype) were excavated in weekly intervals (3 each week, from week 5 to week 12) and imaged in our video box. For both tests, the resulting root diameters were classified according to the schema introduced in [5] with the following class diameter ranges: class 1: <2 mm; class 2: 2-6 mm; class 3: 6-20 mm; class 4: >20 mm. As outlined before, the maximum class of each root indicates the root type (FR, TR, ESR, and SR).

**Figure 7:**
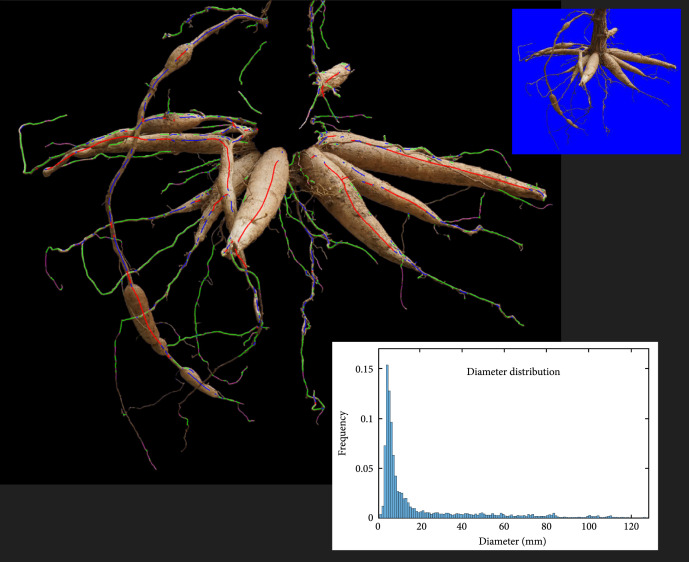
Analyzed image of a fixed cassava root. The stem was removed manually (see top insert for comparison). Colored lines indicate the detected root centers with the following coding: purple-FR, green-TR, blue-ESR, and red-SR. The distribution of root diameters was computed as probabilities with a resolution of 1 mm (see bottom insert).

The image of a fixed cassava root was analyzed with the *reverse* iteration option using the parametrization of Table S1 (center column). Beforehand, the stem was erased from the image manually because for this single image acquired with a different imaging setup (background, illumination, and imaging distance); the stem detection with a retrained Mask R-CNN was comparatively laborious. The detected root center lines, associated classifications and the distribution of root diameters are highlighted in Figure 7. The color-coding reflects the four different diameter ranges. We observe that the majority of roots has been detected with a few exceptions that are featured by small root diameters first. Detected root sections with purple-colored root centers correspond to the tips of various root types (TR, ESR, and SR), while the green-colored sections either indicate a TR or the thinly tapered part of larger roots. Most of the SR contain larger sections of diameter class 4 and smaller sections of diameter class 3, the last one either belonging to the gradually tapered transition region towards the root tips or to misclassified sections within the broader root parts. The noticeable long root with several swollen storage regions (left) nicely displays a mixture of class 3 (blue) and 4 (red) regions, which indicates the transition from ESR to SR in this respective root. We also observe that the two horizontally aligned roots (on the left) are classified as one root at their end and therefore misclassified as class 4 instead of having to roots of class 3.

**Figure 8:**
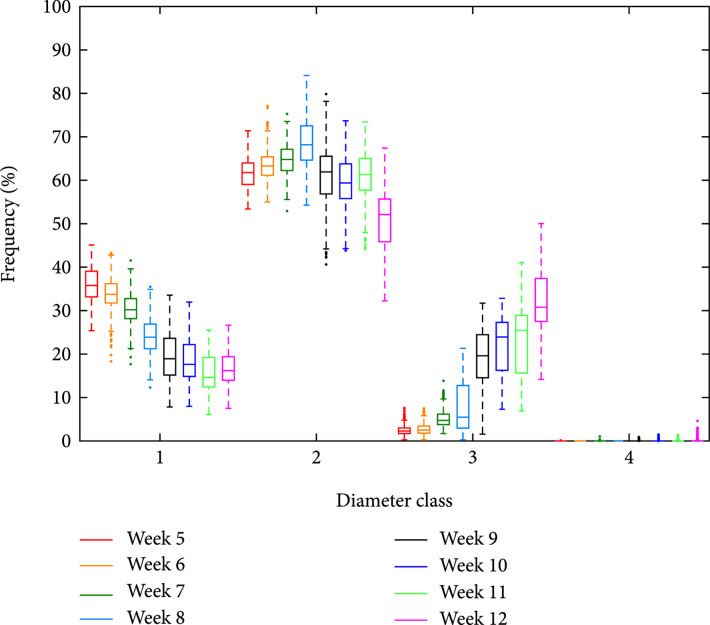
Root class-specific growth dynamics of excavated root systems. Cassava root systems were excavated and analyzed over a time span of 8 weeks. Each week, the results for 3 root systems were used to compute a dispersion statistics, which shows the decline or increase of particular root diameter classes (class 1: <2 mm, class 2: 2-6 mm, class 3: 6-20 mm, and class 4: >20 mm) in the course of time.

In the second experiment, videos of 24 root systems (each with 180 different perspectives captured in regular 2° intervals) were analyzed with the *forward* iteration option and the parameter setting of Table S1 (right). We computed the diameter distributions for each frame and plant separately and rescaled them to percentage portions first. Then, we summed over all frames and plants and calculated the statistical dispersion for each diameter class and excavation date, such that the medians within each excavation week roughly sum up to 100% (Figure 8).

We observe a continuous decline in class 1 diameters over the entire time span, while class 2 diameters first increase up to week 8 before the decline starts. The relevance of this point in time becomes even more apparent, if we look at the sharp increase for class 3 diameters that takes place between week 8 and 9. Thicker roots containing class 4 diameters were not detected yet, however, the occurrence of some outliers in the box plot that increase from week 9 indicates that early stages of storage roots may be present. Instead of using a dispersion statistics (as shown in Figure 8), which averages over all 180 images, there is another option to compute the statistical distribution of diameter classes by considering only those the frames, which display roots of the respective class in the maximally possible way. This can either be achieved by finding the maximum lengths over all frames or by computing a high percentile (e.g., 95%) of the distribution, the latter less error-prone to outliers. Compared to the dispersion statistics, the differences were rather small, and the qualitative conclusion remained unchanged (data not shown). In Figure S2, we give 3 examples for detected root center lines for excavation week 6, 9, and 12. To figure out the impact of the iteration direction on the results, we tested the reverse iteration keeping the parameter setting of Table S1 (right). In comparison to the forward processing option, the diameter classes 1 and 2 were underestimated by 9.7% and 7.8%, while the diameter classes 3 and 4 were overestimated by 7.8% and 9.3%.

## 4. Discussion

This is to our knowledge, the first software based on 2D imaging to follow storage root development to identify phenotypic variation within great plant populations. The software allows the measurement of smaller fibrous roots to large storage roots with several centimeter diameter. We presented three test cases, which entail different challenges to our analysis pipeline and are therefore good examples to illustrate the range of applications of our approach. Images and videos of varying quality served as input data, ranging from artificially rendered roots with perfect surface texture to video frames of real roots that display typical motion effects of the acquisition as well as natural root surface textures. The analysis pipeline can be applied on intact root crowns (both video and image mode) as well as on separated roots (image mode). The second case was not studied here but is supposed to deliver more accurate results due to the absence of overlapping and touching roots. The video mode is a suitable option for high-throughput experiments like the time-series test case, which was sampled from the associated project with the target to phenotype over 54.000 individual plants. Regarding such large-scale projects, the analysis of separated root systems would be too time-consuming. When comparing the known diameter distributions of the simulated root systems with our analysis results, but also when inspecting the real root test cases visually (including e.g. the plausible courses of the analyzed real root growth dynamics), it can be noticed that our approach is capable to deliver good estimations of the diameter distribution even for more complex root systems. Regarding root systems with touching, overlapping or adjoining roots current approaches are either not capable to use the entire image information for root diameter estimations [23, 24] or too costly or elaborate and not high-throughput like tomographic methods [11–13].

### 4.1. Influence of the 2D Imaging Geometry

The 2D approach works best with roots systems that hold enough flexibility such that the majority of roots can be placed in the same plane, while images are taken from a view perpendicular to this exposure. In more rigid root systems with a heterogeneous distribution of single roots (like in our test cases with older cassava plants) imaging from all sides and integrating all analysis delivers reliable results. In 2D space, the number of identified pixels and the computation of the corresponding Euclidian metrics of root center lines depends on two factors. Firstly, it varies with different distances between camera and object. Thus, the roots’ dimensions (lengths and diameters) are enlarged or shortened depending on whether they are positioned before or behind the rotation axis of the system with respect to the camera position. Secondly, all objects are perspectively shortened, if they are not aligned fronto-parallel to the camera. This applies to the length measures. The influence of perspective shortening was not studied with the simulated models, but this issue can be addressed theoretically. The diagram in Figure S3 shows the computed perspective error (percentage shortening) that is introduced by the geometry of our imaging setup. We assumed a pinhole camera model with the imaging parameters of the video box and considered different root inclinations and the averaging effect of the root system rotation. For our setup, we can expect a maximum error of 28.4% for horizontally aligned root sections and an error of 12.7% for a homogeneous distribution of inclination angles. This effect needs to be considered, especially if diameter classes are featured by different inclination angles. The influence of the imaging distance can be reduced, e.g., by having a sufficiently large space between object and camera compared to the object’s expansion. However, the image resolution gets worse with a higher object distance, which (together with the blur effects, discussed later) resulted in a significant loss of detected FR in our study on root growth dynamics. The imaging distance is therefore a trade-off between both factors. For the analysis of the root growth, it was adjusted such that the TR and the resultant emergence of ESR and SR were well detected. For other questions the distance or resolution should be adjusted accordingly taking also other views into account. For example, an optional bottom camera that can be placed underneath shallow root systems. An important biasing factor are blurring effects, which result from the continuous root movement in the video box. This affects primarily the thinnest roots, especially the ones far off the rotation axis. These roots are more strongly blurred and not detected in many cases. Removing them with the blur filter helps to avoid wrong width estimations, which may occur due the enlargement of the diameter due to the motion.

### 4.2. Stem Detection

The stem detection delivered robust results, despite the rather small set of annotated images. The high specificity is most likely a result of the fixed video acquisition setup, where the roots are cut and mounted the same every time and the illumination remains unchanged.

### 4.3. Effects on the Diameter Classes

By using several validation studies of increasing complexity, we could demonstrate the applicability but also some limitations of our root diameter detection and width estimation approach. To better understand the following points, it should be noted that the validation was judged with the distribution of diameter classes, which serve as input parameters of the OSR model, and not pixel wise. The latter variant would be by far more accurate, but could not achieved with the OSR models. The simplest setup, a test with single virtual roots (Figure 3), showed that all root types including the thin FR could be analyzed presuming a sufficiently high image resolution. A few wrong classifications affected higher classes that were not present. They emerged most likely from root sections with diameters close to the class borders. The reduced resolution of the second test with virtual root models of only one class was used to ensure comparability to the video box setup. This has a notable impact on the classification of class 1 diameters such that the thin FR (class 1) is frequently misclassified as class 2 (see Figure 4, left; the same effect is visible in Figure 5(a)). Here, the simulated FR had a diameter of ~3 pixels, which is very close to the class border of (in this case) 3.3 pixels. Regarding the considerable underestimation of diameter class IV (Figure 3, right), it should be noted that this effect emerges most likely due to the overlap of root bases (this effect was observed in every root system with type IV roots, see e.g., Figure 1, F, where two storage roots are overlapping in the connection region to the stem). Both setups with simulated root systems (Figures 3 and 4) display in most cases only slight differences between simulation and ground truth. But it did not display systematic over and underestimation, such that we expect to have reliable predictions for images with a reduced resolution at least for the higher root diameter classes, if enough images of different root systems are acquired.

### 4.4. Software Parametrization

The approach shows a higher sensitivity to three parameters: the contrast levels between roots and background, the iteration number and direction. If a larger range of diameter classes has to be covered three to four iterations seem to be sufficient, as shown in our examples. The choice between *forward* and *backward* iteration direction depends on whether the focus is on the detection of larger roots or not. The *forward* direction that starts with the thinnest roots was the preferred method of choice in most cases, where many FR and TR were present. Here, the *backward* method has the disadvantage that the search starts with the thickest roots by applying a strong blurring on any structure, thereby strongly affecting the small roots and either erasing them completely or, even worse, lead to a misclassification, i.e., a cluster of several small roots could be regarded as one thick root. This effect became apparent in the analysis of 24 physical cassava root systems, where the reverse iteration direction led to an underestimation of the thin diameter classes and an overestimation of the (early) storage roots. In the image example with a single real root, we applied the reverse iteration direction as the smaller root types were only sparsely distributed. In some cases, the classification tends to fail, or roots are not detected at all. False positives that are classified too large have been discussed in the context of the iteration direction. False positives sometimes appear at the border of thicker roots that are classified too small, if an extreme shading is existent or if mixed pixels (of foreground and background) form thin stripes. The first case is also featured by a root center line that is not correctly placed in the center. In some cases, this problem can be solved by increasing the contrast level for these diameter classes. Regarding false negatives, we found that small roots are not detected, when they were too small in diameter (≤ 2 pixels) or motion blurred. In rather rare cases, ESR or SR are also missed, if they are partly covered by small roots (in the *forward* iteration direction), or if they are aligned with another large root, and the contrast between both was too small.

### 4.5. Transferability

Although we chose cassava as a model species as it is an economically important storage root crop, we envision that the software could be applied to other root system with similar root morphology such as sweet potato, yams, taro, etc. The developed software enables us to phenotype of a great number of plants and, subsequently, measure phenotypic variations. The correlation of phenotypic and genotypic variation enables to the identification of quantitative traits loci/genes that are putative involved in storage root development. Subsequently, the identifications of those loci/genes are going to be the basis for future plant breeding, targeting storage root formation and, subsequently, food security for the tropical and subtropical regions.

## Data Availability

The data presented in this study are openly available in Zenodo.org (doi:10.5281/zenodo.5883368) [39]. The software has been published in Gitlab under https://gitlab-public.fz-juelich.de/grow-screen-field. The parameters of the OSR-models are available from the authors upon request.

## Supplementary Materials

Supplementary Materials Figure S1. Regression statistics of the validation experiments Figure S2. Analyzed real roots examples for root systems of different developmental stages Figure S3. Error estimation from perspective shortenings Table S1. Parameter constellation for the analysis of simulated and real root images.
