# Supplementary material for: Assessing the Storage Root Development of Cassava with a New Analysis Tool
Source: Plant Phenomics. 2022 Oct 26;2022:9767820. doi: 10.34133/2022/9767820 (PMC10204708; doi:10.34133/2022/9767820)
Supplement: Supplementary Materials — Figure S1. Regression statistics of the validation experiments Figure S2. Analyzed real roots examples for root systems of different developmental stages Figure S3. Error estimation from perspective shortenings Table S1. Parameter constellation for the analysis of simulated and real root images. [file 9767820.f1.docx]

Supplementary Figures


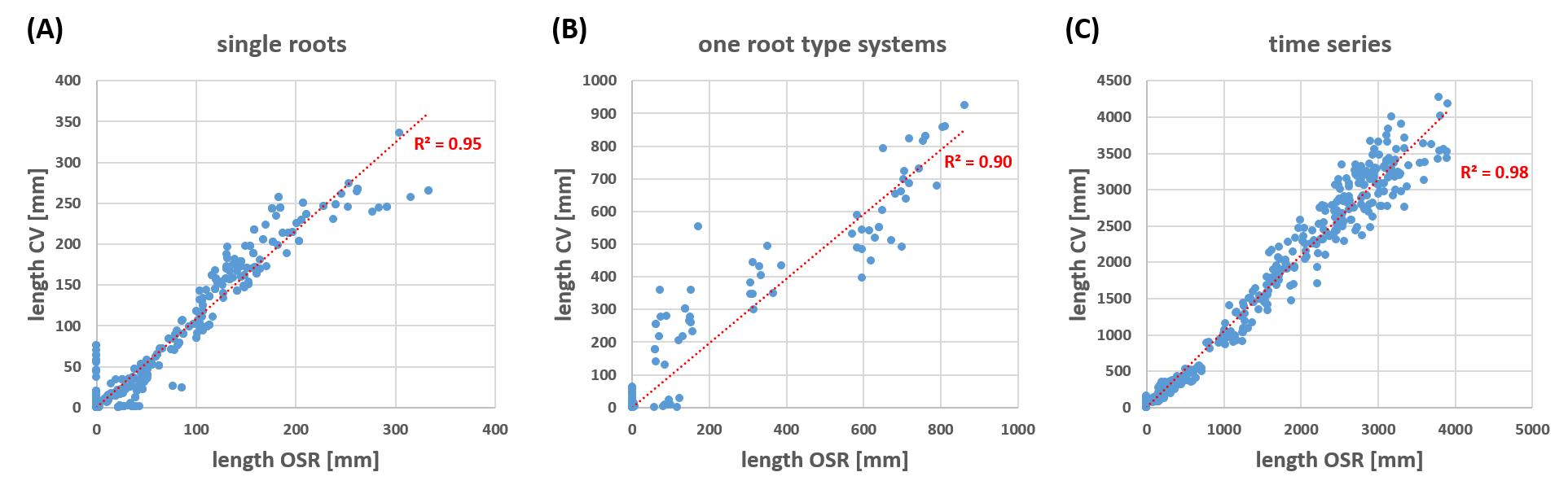


Figure S1. Regression statistics of the validation experiments. In the diagrams compare the constituting lengths for the different root diameter classes of the OpenSimRoot (OSR) models with the estimations from the computer vision (CV) pipeline and computed the coefficients of determination (R²) for each experiment. (A) In the single root models (800 data points) we achieved a high correlation of $\boldsymbol{R}^{\boldsymbol{2}}\boldsymbol{=0.95}$ (nRSME = 0.27); (B) in the models that contained several roots of only one root type (140 data points, not considering root type I) the estimations were slightly weaker with an $\boldsymbol{R}^{\boldsymbol{2}}\boldsymbol{=0.9}$ (nRSME = 0.31); (C) the highest correlation was achieved in the time series (900 data points, not considering root type I) and $\boldsymbol{R}^{\boldsymbol{2}}\boldsymbol{=0.98}$ (nRSME = 0.15).


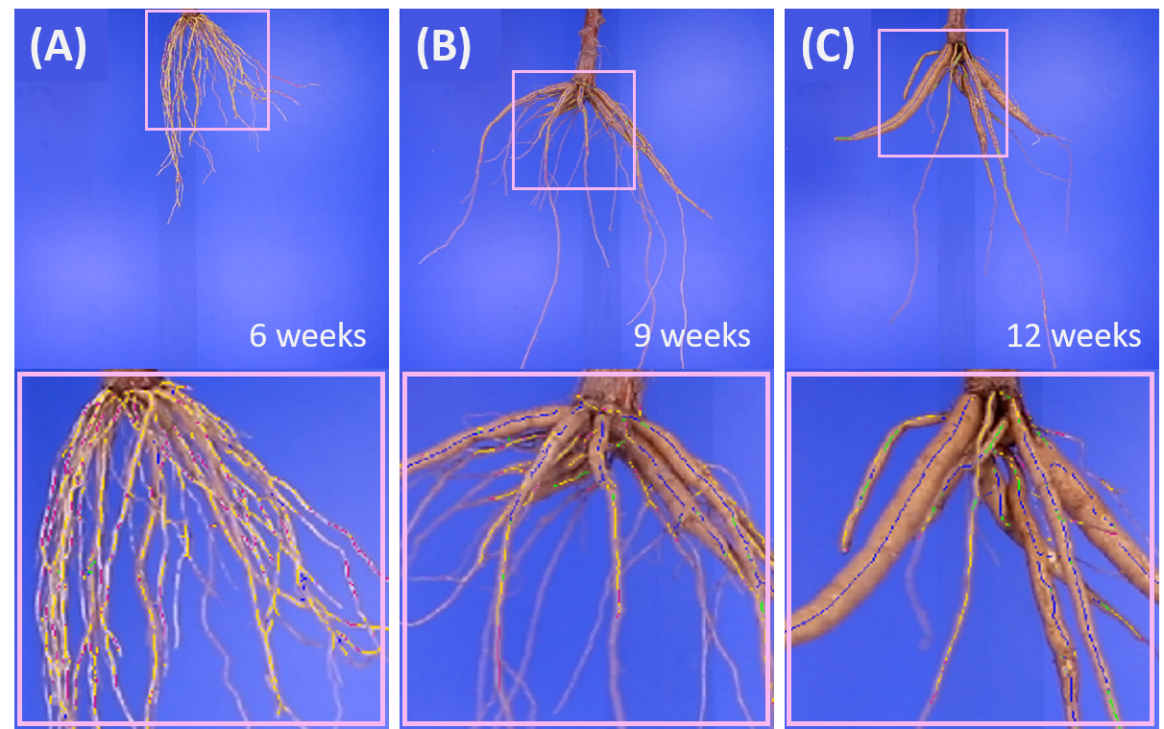


**Figure S2. Analyzed real roots examples for root systems of different developmental stages**. From the 24 root systems that were analyzed in weekly intervals we give 3 examples for week 6, 9 and 12 (each with one frame) that illustrate the transition from lower root diameter classes (1 and 2) to higher classes (2 and 3). Class 4 root diameters hardly occurred in this test case. The color-coding corresponds to the root diameter classes. The color-coded lines indicate the central root axis (purple-class 1, green-class 2, blue-class 3, class 4 was not detected). FR with strong blurring were not detected. (**A**) Week 6 root systems displays a mixture of class 1 and 2 detections. (**B**) Week 9 roots started the transformation from TR (green) to ESR (blue). (**C**) Week 12 roots display some enlarged ESR that are close to the transformation to SR.


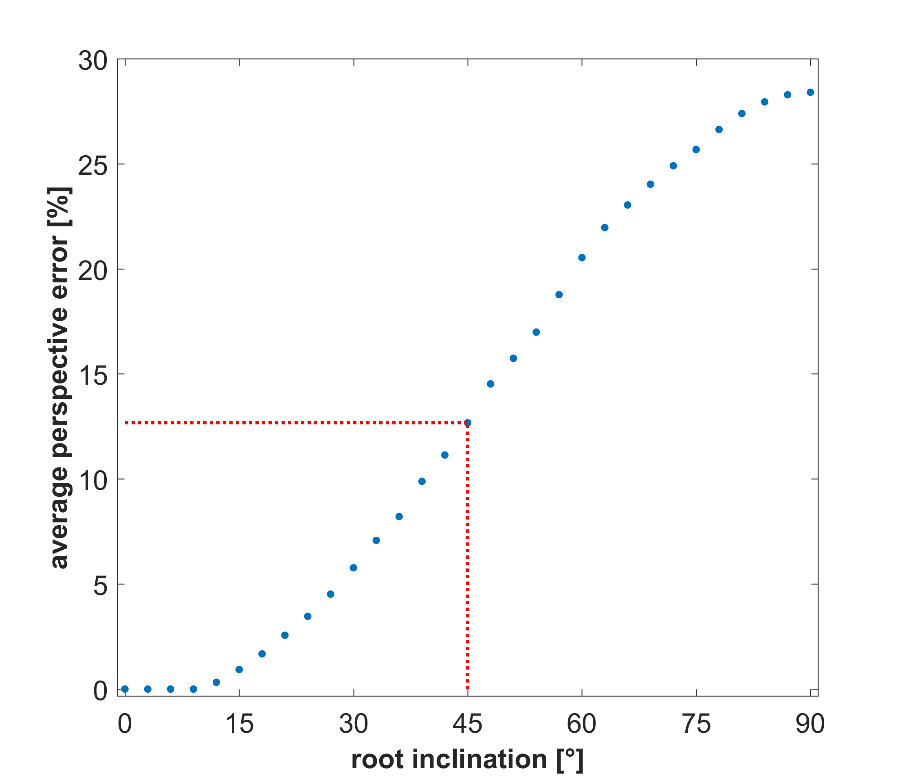


**Figure S3. Error estimation from perspective shortenings.** The error was computed by assuming a 360° rotation (in 2° steps) of straight roots that were inclined from 0° (vertical) to 90° (horizontal). The error was computed by assuming a pinhole projection with the imaging geometry of our setup (imaging distance 1135 mm, focal length 35 mm) and by averaging over all 180 perspectives. The maximum error of 28.4% occurs with a horizontal root. For a homogeneous distribution of root angles, one would expect an error of 12.7% (red line).

Supplementary Tables

**Table S1. Parameter constellation for the analysis of simulated and real root images.** We applied different parameter constellation for the analysis of virtual and real root image and video data due to the different nature of virtual and real images. Table S1 gives a summary of the three principal parametrizations. One key difference lies in a higher erosion value$\theta$ for real images (in the root segmentation module) mainly due to the fact that the transition region between root and background consists of many mixed pixels showing both foreground and background properties. The other one affects the number of iterations and a slight adaption of $l$ and γ (in the root width analysis). We parametrized each iterations according to the root diameter ranges of 0-3mm, 3-11mm, 11-50mm for OSR models and 0-3mm, 3-8mm, 8-16mm, 16-50mm for real images. We used $\sigma=w/\sqrt{3}$ and assumed a fixed contrast $h_{OSR}=55$ for OSR models and variable contrasts $h_{RR}=var$ for real images (*_rev* refers to the *reverse* iteration direction applied for the single image of a fixed root; *_forw* refers to the *forward* iteration direction for the analysis of video frames). Parameter$u$ was calculated from equation 5. For all iterations we used$l=0.5u$. For the computation of the dilation mask D we applied$\gamma=\left\lceil3w \right\rceil.$

|  | para-meter | validation -OSR models (*forw.*) | real root (RR) single image (*rev.*) | real root (RR) videos (*forw.*) |
| --- | --- | --- | --- | --- |
| ***Root Segmentation*** | | | | |
| segmentation | T_β_ | 80 | 210 | 80 |
| post-processing | θ | 1 | 3 | 3 |
|  | T_F_ | 25 | 25 | 25 |
| ***Root Width Analysis*** | | | | |
| iteration 1 | *σ_1_* | 0.96 | 30 | 0.96 |
|  | *u_1_* | 21.92 | 0.05 | 23.92 |
| *h_OSR_* = 55 | *l_1_* | 10.96 | 0.025 | 11.96 |
| *h_RR_rev_* = 80 | *γ_1_* | 5 | - not used - | 5 |
| *h_RR_forw_* = 60 | *τ_1_* | - not used - | 29 | - not used - |
| iteration 2 | *σ_2_* | 3.53 | 20 | 2.57 |
|  | *u_2_* | 1.64 | 0.066 | 2.52 |
| *h_OSR_* = 55 | *l_2_* | 0.82 | 0.033 | 1.26 |
| *h_RR_rev_* = 85 | *γ_2_* | 17 | 45 | 12 |
| *h_RR_forw_* = 45 | *τ_2_* | - not used - | 17 | - not used - |
| iteration 3 | *σ_3_* | 16.04 | 10 | 5.13 |
|  | *u_3_* | 0.08 | 0.26 | 0.5 |
| *h_OSR_* = 55 | *l_3_* | 0.04 | 0.13 | 0.25 |
| *h_RR_rev_* = 100 | *γ_3_* | - not used - | 31 | 24 |
| *h_RR_forw_* = 35 | *τ_3_* | - not used - | 11 | - not used - |
| iteration 4 | *σ_4_* | - not used - | 2.5 | 16.04 |
|  | *u_4_* | - not used - | 5 | 0.036 |
| *h_RR_rev_* = 125 | *l_4_* | - not used - | 2.5 | 0.018 |
| *h_RR_forw_* = 25 | *γ_4_* | - not used - | 11 | - not used - |
|  | *τ_4_* | - not used - | 3 | - not used - |
